# Development of pulmonary bronchiolo-alveolar adenocarcinomas in transgenic mice overexpressing murine c- *myc* and epidermal growth factor in alveolar type II pneumocytes

**DOI:** 10.1054/bjoc.2000.1676

**Published:** 2001-03

**Authors:** A Ehrhardt, T Bartels, A Geick, R Klocke, D Paul, R Halter

**Affiliations:** Center for Medical Biotechnology, Fraunhofer Institute for Toxicology and Aerosol Research, Nikolai-Fuchs-Str. 1, Hannover, 30625, Germany; Department of Pediatrics and Genetics, Stanford University School of Medicine, 300 Pasteur Drive, Stanford, CA 943005, USA; Institute of Veterinery Pathology, Freie Universität Berlin, Berlin, 14163, Germany; Dr. Margarete Fischer-Bosch-Institut für Klinische Pharmakologie Auerbachstr., 112D-70376 Stuttgart, Germany; Ingenium Pharmaceuticals AG, Lochhamer Str. 29, Martinsried, 82152, Germany

**Keywords:** c-*myc*, alveolar type II pneumocytes, adenocarcinoma

## Abstract

Transgenic mouse models were established to study tumorigenesis of bronchiolo-alveolar adenocarcinomas derived from alveolar type II pneumocytes (AT-II cells). Transgenic lines expressing the murine oncogene c- *myc* under the control of the lung-specific surfactant protein C promoter developed multifocal bronchiolo-alveolar hyperplasias, adenomas and carcinomas respectively, whereas transgenic lines expressing a secretable form of the epidermal growth factor (IgEGF), a structural and functional homologue of transforming growth factor α (TGFα), developed hyperplasias of the alveolar epithelium. Since the oncogenes c- *myc* and TGFα are frequently overexpressed in human lung bronchiolo-alveolar adenocarcinomas, these mouse lines are useful as models for human lung bronchiolo-alveolar adenocarcinomas. The average life expectancies of hemizygous and homozygous c- *myc* transgenics were 14.25 months and 9.2 months, respectively, suggesting that a dosage effect of c- *myc* caused an accelerated bronchiolo-alveolar adenocarcinoma formation. First analyses of double transgenics, hemizygous for both c- *myc* and IgEGF, show that these mice develop bronchiolo-alveolar adenocarcinomas at the average age of 9 months, indicating that these oncogenes cooperate during the lung cancer formation. Our results demonstrate that c- *myc* and EGF are directly involved and cooperate with one another during formation of bronchiolo-alveolar adenocarcinomas in the lung. © 2001 Cancer Research Campaign http://www.bjcancer.com


*British Journal of Cancer* (2001) **84**(6), 813–818
© 2001 Cancer Research Campaign
doi: 10.1054/ bjoc.2000.1676, available online at http://www.idealibrary.com on IDEAL®

http://www.bjcancer.com

### Development of pulmonary bronchiolo-alveolar adenocarcinomas in transgenic mice overexpressing murine c-*myc* and epidermal growth factor in alveolar type II pneumocytes

**A Ehrhardt[1,2], T Bartels[3], A Geick[1,4], R Klocke[1,5], D Paul[1,5] and R Halter[1]**

[1]Center for Medical Biotechnology, Fraunhofer Institute for Toxicology and Aerosol Research, Nikolai-Fuchs-Str. 1, 30625 Hannover, Germany; [2]Department of Pediatrics and Genetics, Stanford University School of Medicine, 300 Pasteur Drive, Stanford, CA 943005, USA; [3]Institute of Veterinery Pathology, Freie Universität Berlin, 14163 Berlin, Germany; [4]Dr. Margarete Fischer-Bosch-Institut für Klinische Pharmakologie Auerbachstr. 112, D-70376 Stuttgart, Germany; [5]Ingenium Pharmaceuticals AG, Lochhamer Str. 29, 82152 Martinsried, Germany

**Summary** Transgenic mouse models were established to study tumorigenesis of bronchiolo-alveolar adenocarcinomas derived from alveolar type II pneumocytes (AT-II cells). Transgenic lines expressing the murine oncogene c-*myc* under the control of the lung-specific surfactant protein C promoter developed multifocal bronchiolo-alveolar hyperplasias, adenomas and carcinomas respectively, whereas transgenic lines expressing a secretable form of the epidermal growth factor (IgEGF), a structural and functional homologue of transforming growth factor α (TGFα), developed hyperplasias of the alveolar epithelium. Since the oncogenes c-*myc* and TGFα are frequently overexpressed in human lung bronchiolo-alveolar adenocarcinomas, these mouse lines are useful as models for human lung bronchiolo-alveolar adenocarcinomas. The average life expectancies of hemizygous and homozygous c-*myc* transgenics were 14.25 months and 9.2 months, respectively, suggesting that a dosage effect of c-*myc* caused an accelerated bronchiolo-alveolar adenocarcinoma formation. First analyses of double transgenics, hemizygous for both c-*myc* and IgEGF, show that these mice develop bronchiolo-alveolar adenocarcinomas at the average age of 9 months, indicating that these oncogenes cooperate during the lung cancer formation. Our results demonstrate that c-*myc* and EGF are directly involved and cooperate with one another during formation of bronchiolo-alveolar adenocarcinomas in the lung.
© 2001 Cancer Research Compaign http://www. bjcancer.com

**Keywords**: c-*myc*; alveolar type II pneumocytes; adenocarcinoma

Pulmonary tumours can be classified as either small cell lung carcinomas (SCLCs) or non-small cell lung carcinomas (NSCLCs). About 110 000 lung cancer patients in the United States per year were killed by NSCLCs, which represent approximately 75% of all lung tumours (Minna et al. 1989) including large cell carcinomas, squamous cell carcinomas and bronchiolo-alveolar adenocarcinomas (synonyms: adenocarcinoma, alveolar cell carcinoma). It is currently estimated that 30–40% of all human lung tumours are adenocarcinomas derived from either alveolar type II pneumocytes (AT-II cells), Clara cells of the bronchiolar epithelium or from mucin-producing cells (Tuveson and Jacks, 1999). AT-II cells and Clara cells secrete pulmonary surfactant, a complex mixture of phospholipids and surfactant proteins (SP) (Weaver and Whitsett, 1991). The function of the 4 known surfactant proteins SP-A, SP-B, SP-C and SP-D is to contribute to the reduction of surface tension in the lung and to facilitate gas exchange. The expression of SP-C is restricted to AT-II cells whereas all other surfactant proteins are secreted by both AT-II and Clara cells (reviewed in Weaver and Whitsett, 1991; Korfhagen et al, 1992). Therefore, the promoter regions of surfactant protein genes are appropriate candidates for use in the construction of lung specific gene constructs.

*Received 18 April 2000*
*Revised 19 December 2000*
*Accepted 19 December 2000*

*Correspondence to:* R Halter

Several proto-oncogenes, including c-*myc* and the transforming growth factor α (TGFα) as well as its homologue, epidermal growth factor (EGF), are frequently found to be overexpressed in human pulmonary carcinoids and adenocarcinomas (Battista et al, 1993; Broers et al, 1993; Lorenz et al, 1994; Moody, 1996), suggesting that they may be directly involved in lung carcinoma formation. c-myc is a member of a group of regulatory proteins which are involved in controlling cell cycle entry, progression and differentiation (reviewed in Facchini and Penn, 1998).

The EGF family includes EGF, TGFα and heparin-binding EGF. Both EGF and TGFα bind to and activate the EGF-receptor (EGFR) (Yeh and Yeh, 1989). Bronchiolo-alveolar adenocarcinomas often show constitutive overexpression of EGFR as well as of TGFα, which indicates that the resulting autocrine loop promotes loss of cell cycle control (Tateishi et al, 1990). The oncogenic potential of these growth factors is supported by the observation that overexpression of TGFα or EGF in the liver of different transgenic mouse strains cause hepatocellular carcinomas (Sandgren et al, 1993; Tönjes et al, 1995).

In this work we used the AT-II cell specific SP-C promoter to generate transgenic mouse lines constitutively overexpressing the oncogene c-*myc* and a secretable form of EGF (IgEGF) (Tönjes et al, 1995) in the lung. Transgenics expressing c-*myc* developed multifocal bronchiolo-alveolar adenomas and carcinomas respectively, those expressing IgEGF developed multifocal alveolar hyperplasias. Cooperation in lung tumour formation of both transgenes was demonstrated in IgEGF/*myc* double transgenic mouse lines. The established transgenics will provide useful animal models to test targeted gene therapy protocols, in which the expression of potentially cytotoxic gene products can be targeted to cancer cells by the SP-C promoter.

### MATERIALS AND METHODS

### Cloning procedures and production of transgenic mouse lines

The *Apa*I-*Hind*III mouse c-*myc* DNA fragment from the plasmid pTG2948 (Dalemans et al, 1990) was subcloned into the corresponding restriction sites in pBSKS II (+/–) (Stratagene). *Apa*I was converted into a *Sal*I restriction site. The 2.7 kb *Sal*I/*Eco*RI *c-myc* DNA fragment was ligated to the *Sal*I/*Eco*RI site of the vector pUC18/3.7SP-C downstream of the human SP-C promoter 5′-flanking region (Wikenheiser et al, 1992). The *Bam*HI site of the *Bam*HI–*Sal*I IgEGF fragment (nucleotides 0 to 360, including the Ig signal sequence and a synthetic EGF gene) derived from the plasmid alb-DS4 (Tönjes et al, 1995) was converted to a *Sal*I restriction site. The new IgEGF *Sal*I fragment was cloned into the *Sal*I restriction site 3′ of the promoter of the human SP-C gene of pUC18/3.7SP-C. Both gene constructs were cleaved with *Nde*I and *Not*I and the fusion gene fragments were purified by the Qiagen gel extraction kit and microinjected into male pronuclei of fertilized oocytes from hybrid CD2/F1 (DBA/2 × Balb/C) mice (Hogan et al, 1994). Viable oocytes were transferred into the oviduct of pseudopregnant CD2F1 recipient mice. Transgenic founder mice were mated with CD2F1 for propagation as hemizygous transgenics.

### Southern and Northern analysis

Transgenic mouse lines were identified by Southern analysis of DNA extracted from biopsied mouse tails (Hogan et al, 1994). Restricted DNA was separated through 0.8% agarose and transferred to nylon membrane (Amersham Life Sciences) according to standard protocols. Hybridization was performed in Church buffer (0.25 M $NaHPO_4$, 7.0% SDS, 10 mM EDTA, pH 7.2) at 65˚C with the randomly labelled transgene.

Total RNA from various tissues was isolated by the Qiagen RNA extraction kit after homogenization using a Polytron homogenizer and blotted according to standard protocols.

### Histopathology

Tissues were fixed in 4% paraformaldehyde in PBS for approximately 20 h, dehydrated and embedded in paraffin (Roti®-Plast, Roth). Tissue sections were stained with haematoxylin & eosin according to standard protocols. The mouse tumours were classified according to the International Agency for Research on Cancer (IARC) – WHO (2000).

### Reverse slot blot hybridization

To analyse gene expression in lung adenocarcinomas by reverse slot blot hybridization, 6 micrograms of each recombinant cDNA clone were blotted onto nylon-reinforced nitrocellulose (Schleicher & Schuell). Total RNA was isolated from different lung tissues using the Qiagen RNA easy kit. Synthesis and labelling of single strand cDNA was performed as follows: 25–50 µg total RNA were dissolved in 17 µl $H_2O$ and incubated with 5 µl oligo $(dT)_{12–18\ bp}$ (0.5 mg $ml^{-1}$) at 70˚C for 10 min. 1.5 µl RNasin (10 U $\mu l^{-1}$), 7.5 µl dNTP-mix (ATP, TTP and GTP each, 5 mM), 3 µl dCTP (0.27 mM), 15 µl 5 × reverse transcriptase reaction buffer, 7.5 µl 0.1 M DTT, 15 µl α-[$^{32}$P]-dCTP and 3.75 µl Superscript reverse transcriptase (200 U $\mu l^{-1}$) (Life Technologies) was added and incubated at 42˚C for 1 h. Free nucleotides were removed with Micro Bio-Spin 6 Chromatography Columns (BioRad). RNA/cDNA hybrids were denatured with one volume of 0.3 N NaOH, 30 mM EDTA and boiled for 5 min, chilled on ice and neutralized with 0.5 volume of 1M Tris-CL, pH 8. Hybridization of slot blot nitrocellulose filters was performed in Church buffer for 24 h at 65˚C. After washing under stringent conditions (0.1% SDS; 0.1 × SSC; 65˚C) for at least 30 min, autoradiography was performed with Kodak XOmat AR X-ray film.

The following c-DNA-probes were used: actin, c-DNA/murine β actin; control plasmid, pBR322; SP-A, c-DNA/murine SP-A; SP-B, c-DNA/murine SP-B; SP-C, c-DNA/murine SP-C; cycD1, c-DNA/human cyclin DI; cdc2, c-DNA/human cyclin dependent kinase; *c-jun*, c-DNA/ murine transcription factor *c-jun*.

### RESULTS

### Generation of transgenic mouse lines and their phenotypes

The gene constructs SP-C/*myc* and SP-C/IgEGF (Figure 1A, B) consisted of the murine c-*myc* gene and a secretable form of EGF (IgEGF), whose expression were controlled by the human SP-C promoter. One SP-C/IgEGF and 5 SP-C/*myc* founder mice were identified by the generation of diagnostic fragments upon restriction enzyme digestion of mouse tail DNA and subsequent Southern analysis as shown representative for SPC/*myc* transgenic mice in Figure 3. Transgenic mouse lines were established from the SP-C/IgEGF and two SP-C/*myc* transgenic founder mice. All other founder mice were not germ line transgenic and did not transfer the transgene to their descendants. 2 of the SP-C/*myc* founder mice showed hyperplasias in the lung alveolar epithelium (not shown). In contrast, the founder SP-C/*myc* 3.2 as well as all established transgenic mouse lines, e.g SP-C/*myc* 8.2 and 13.0, developed multifocal pulmonary bronchiolo-alveolar adenocarcinomas originating from the alveolar epithelium. Littermates of the SP-C/IgEGF transgenic mouse line 6.2 showed no bronchiolo-alveolar adenocarcinomas, but they developed hyperplasias derived from the alveolar epithelium. The observed phenotypes of all founder mice and their offspring are summarized in Table 1. Transgene expression could be detected in the lung from all shown founder animals and the copy number of the transgene c-*myc* was 1–2 copies for the established transgenic lines SPC/myc 8.2 and 13.0 and 2–3 copies for the founder animals SPC/myc 3.2 and SPC/myc 13.0 (Table 1). The following work is focused on the transgenic mouse lines SP-C/*myc* 8.2 and SP-C/IgEGF 6.2.

### Expression of the SP-C/*myc* and SP-C/IgEGF transgene

RNA was isolated from tissues of littermates of the transgenic mouse lines SP-C/*myc* 8.2 and SP-C/IgEGF 6.2 and subjected

 *© 2001 Cancer Research Campaign*

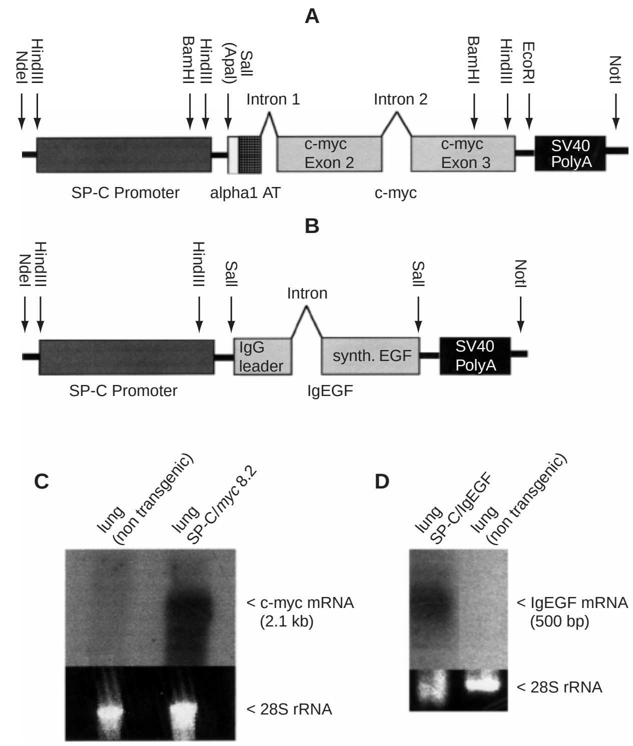

**Figure 1** Fusion genes SP-C/*myc* **(A)** and SP-C/IgEGF **(B)** for generation of transgenic mice. Northern analysis of total RNA from lung tissue of SP-C/*myc* **(C)** and SP-C/IgEGF **(D)** transgenic mice. The 2700 bp *Sal*I/*Eco*RI c-myc and the 360 bp *Sal*I IgEGF fragments were used as [$^{32}$P] labelled probes for hybridization in Northern analysis.

to Northern analysis. c-*myc*- and IgEGF-specific mRNAs were detected only in the lung of both transgenic mice (Figure 1C, D) and not in any other tissue including salivary gland, liver, pancreas and ovary (not shown). Non-transgenic mice showed no signal in the lung for both transgenes, respectively (Figure 1C, D).

### Development of hyperplasias in SP-C/IgEGF transgenics and development of bronchiolo-alveolar adenocarcinomas in SP-C/myc transgenics

Expression of the SP-C/IgEGF transgene induced the development of alveolar hyperplasias in the alveolar epithelium (Figure 2A) when compared to non-transgenic mice (Figure 2B). Alveolar hyperplasias in analysed SPC/IgEGF individuals occurred at the average of 19 months. In SP-C/*myc* transgenics different stages of tumour development in the alveoli were frequently observed. Large bronchiolo-alveolar adenocarcinoma developed only in the lung of SP-C/*myc* transgenics. Early stages of tumour development were characterized by multifocal hyperplasias originating in the alveolar epithelium (Figure 2C). Adenomas, which developed in the alveolar septae were observed in lung sections of SP-C/*myc* transgenics at the age of 6–7 months (Figure 2D). Advanced stages of carcinogenesis consisted of multifocal bronchiolo-alveolar adenocarcinomas (Figure 2E) were detected in SP-C/*myc* transgenics at the average age of 14.25 months, whereas the bronchiolar epithelium was not affected. Figure 2F demonstrates a lung of a non-transgenic and of a SP-C/*myc* transgenic mouse, both of 14 months of age. One lobe of the lungs was completely transformed to a bronchiolo-alveolar adenocarcinoma.

### Generation of homozygous SP-C/*myc* transgenics and hemizygous double transgenic mice expressing c-*myc* and IgEGF

The medial survival times of hemizygous SP-C/*myc* transgenics is 14.25 months (Table 2), whereas the medial age of death of homozygous SP-C/*myc* transgenic is 9.2 months (Table 2). At the age of 14.25 months and 9.2 months respectively, 75% of all hemizygous and 80% of homozygous mice were diagnosed with bronchiolo-alveolar adenocarcinomas transforming both lung lobes (Table 2 and Figure 2F). These findings suggest that a gene dosage effect of c-*myc* expression contributed to the accelerated tumor development as compared to hemizygous transgenics. Hemizygous and homozygous mice were distinguished by Southern analysis (Figure 3). A summary of homozygous and hemizygous SP-C/*myc* transgenics, their phenotype, and their life expectancies are shown in Table 2. These results demonstrated that c-*myc* overexpression was causally involved in bronchiolo-alveolar adenocarcinoma formation. A gene dosage effect was also observed in another transgenic mouse, who expressed SV40 Tag under the control of the fetal globin promoter. In this transgenic mouse strain prostate tumours were induced in 75% of the male hemizygous for the transgene but in 100% of all male homozygous mice (Perez-Stable et al, 1997).

**Table 1** Summary of examined SP-C/*myc* and SP-C/IgEGF transgenic founder mice, their offspring and their phenotypes

| Founder | transgenic line [No. of generations] | Transgene expression (lung)/ copy number of the transgene | Phenotypes in the lung |
|---|---|---|---|
| SP-C/*myc* 3.2 | no | +/2–3 | Multifocal bronchiolo-alveolar adenomas and bronchiolo-alveolar adenocarcinomas |
| SP-C/*myc* 4.2 | no | +/2–3 | Hyperplasias in alveolar epithelium |
| SP/C/*myc* 8.2 | yes [20] | +/1–2 | Multifocal bronchiolo-alveolar adenomas and brochiolo-alveolar adenocarcinomas |
| SP-C/*myc* 13.0 | yes [7] | +/1–2 | Multifocal brochiolo-alveolar adenomas and bronchiolo-alveolar adenocarcinomas |
| SP-c/*myc* 16.2 | no | +/2–3 | Hyperplasias in alveolar epithelium |
| SP-C/IgEGF 6.2 | yes [20] | +/n.d | Hyperplasias in alveolar epithelium |

© 2001 Cancer Research Campaign

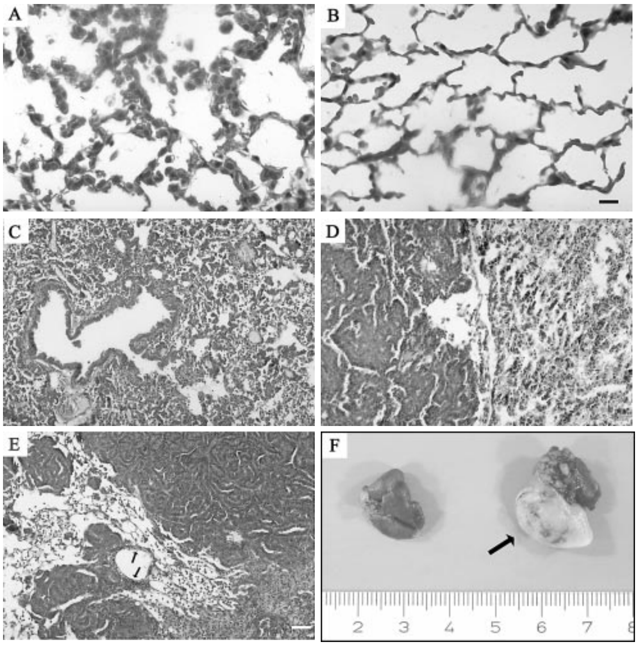

**Figure 2** Histology of a lung of a non transgenic and a SP-C/IgEGF transgenic mouse. **(A)** The lung of a SP-C/IgEGF transgenic mouse was showing multifocal hyperplasias of the alveolar epithelium which was indicated by the increased cellularity in the alveoli. **(B)** A normal lung with thin alveolar septae in a non transgenic mouse. (A, B: bar = 20 μm). Several stages of tumor development frequently observed in the lungs of the transgenic mouse line SP-C/*myc* 8.2. **(C)**, alveolar hyperplasia: The alveoli exhibit a hyperplastic epithelium consisting of cuboidal cells lining the alveolar septa and ducts. **(D)**, bronchiolo-alveolar adenoma: It is indicated by a circumscript neoplasia forming papillary patterns (right of the picture). The surrounding lung tissue is compressed by the tumor growth (left). **(E)**, bronchiolo-alveolar adenocarcinoma: Cells invading the alveoli exhibit a papillary growth pattern, whereby the bronchiolus (arrows) is not affected. Due to the invasion of tumor cells in the adjacent lung tissue the tumors appear poorly circumscribed. Note the multifocal origin of the bronchiolo-alveolar adenocarcinoma. Progressive growth resulting finally in solitary tumor masses replacing the lung parenchyma. (C–E: bar = 200 μm). **(F)** Lung of a SP-C/*myc* transgenic and of a non transgenic mouse. The lung of the SP-C/*myc* transgenic mouse developed bronchiolo-alveolar adenocarcinoma replacing most of the normal lung tissue (arrow).

To generate hemizygous double transgenics expressing c-*myc* and IgEGF, offspring of transgenic mouse lines SP-C/*myc* 8.2 and SP-C/IgEGF 6.2 were cross-bred. Littermates were analysed for the presence of both transgenes by Southern analyses. The life expectancy of SP-C/*myc*/IgEGF double transgenic individuals analysed so far was 9 months, which was clearly reduced compared to the medial survival times of hemizygous SP-C/*myc* or SP-C/IgEGF transgenics, i.e. 14.25 and 19 months, respectively (Table 2). 100% of examined SP-C/*myc*/IgEGF double transgenics were diagnosed with bronchiolo-alveolar adenocarcinomas (Table 2). From these results we conclude, that c-myc and IgEGF cooperated during lung tumour formation. Histological analysis confirmed that lung tumours in SP-C/*myc*/IgEGF double transgenics originated from AT-II cells and thus were classified as bronchiolo-alveolar adenocarcinomas (not shown). The number of lesions in the 4 examined double transgenics is macroscopically lower but the lesion size is enlarged in comparison to *SPC/myc* transgenics at the age of 9 months. We speculate that additional randomly occurring genetic changes in each lesion have to take place for tumour induction.

### Gene expression in lung tumours of transgenic mice

Expression of one transgene in a target tissue is usually not sufficient for tumour development. Therefore we analysed tumours for abnormal expression of selected proto-oncogenes. We also checked expression patterns of genes which are known to be typically expressed in AT-II cells, in order to obtain information about the extent of dedifferentiation of the tumour cells as compared with AT-II cells from which they were derived. For this purpose we used the reverse Northern slot blot hybridization technique. Lungs of non-transgenic CD2F1 littermates (14 months old), from tumour nodules of SP-C/*myc* transgenics (14 months old) and from tumour nodules of SP-C/*myc*/IgEGF double transgenics (9 months old) were investigated. The intensity of mRNA expression of three surfactant proteins, which are known to be expressed in AT-II cells, differed moderately among the analysed tissues in comparison to those of non-transgenic littermates (Figure 4). The expression levels also differed among tissues from mice expressing c-*myc* or both, c-*myc* and IgEGF, which may indicate various stages of dedifferentiation of the lung tumour tissue.

To analyse the expression levels of genes involved in regulating cell proliferation we analysed the expression of selected cell cycle regulating genes including cyclin D1, cdc2 and c-*jun*. The

**Table 2** Summary of analysed homozygous and hemizygous mice and their pheotypes; (+/0), hemizygous mice; (+/+), homozygous mice

| Transgenic mouse line | Genotype | Pathology | % of animals with each kind of pathology [No. of analysed animals] | Medical age of Death [mean values +/–SEM] |
|---|---|---|---|---|
| SP-C/myc 8.2 | +/0 | bronchiolo-alveolar adenocarcinomas | 75% [14] | 14.25 +/–1.07 months |
| SP-C/IgEGF | +/0 | Hyperplasia | 66.7% [3] | 19 +/–0.41 months |
| SP-C/myc 8.2 | +/+ | bronchiolo-alveolar adenocarcinomas | 80% [5] | 9.2 +/–1.07 months |
| SP-C-myc/IgEGF | +/0; +/0 | brochiolo-alveolar adenocarcinomas | 100% [4] | 9.0 +/–1.29 months |

© 2001 Cancer Research Campaign

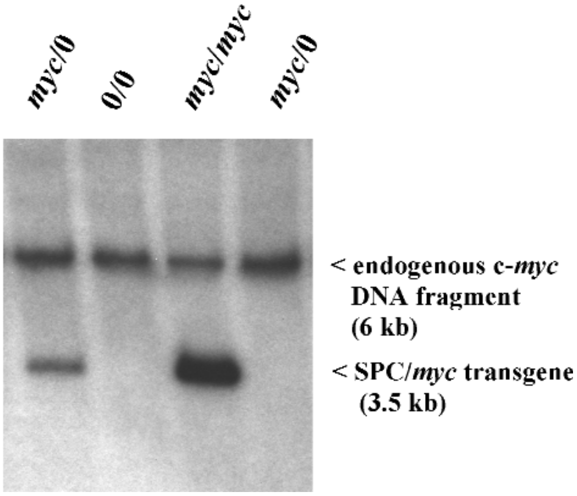

**Figure 3** Southern analysis of homozygous and hemizygous SP-C/*myc* 8.2 transgenic mice. DNA, isolated from biopsied tails of SP-C/*myc* transgenic mice, was digested with *Bam*HI. A diagnostic 3.5 kb transgene specific fragment was detected in the DNA of all transgenic mice but not in non transgenic mice. An additional 6.0 kb *Bam*HI fragment represents the endogenous *c-myc* DNA fragment, which was found in transgenic and non transgenic mice. Homozygous transgenic mice are characterized by a stronger signal of the transgene specific c-*myc* DNA fragment in comparison to the endogenous c-*myc* DNA fragment. *myc*/0, hemizygous SP-C/*myc* transgenic mouse; *myc/myc*, homozygous SP-C/*myc* transgenic mouse; 0/0, non transgenic mouse

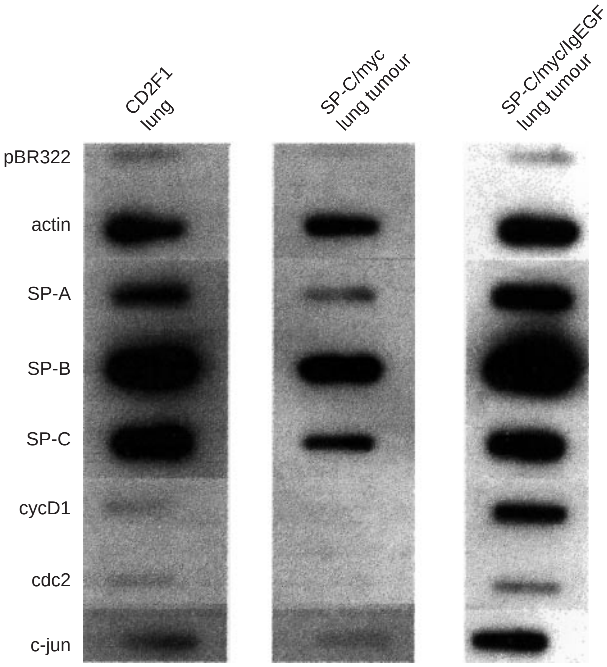

**Figure 4** Gene expression profiles in the lungs of non transgenic littermates, in SP-C/*myc* transgenic and in SP-C/*myc*/IgEGF double transgenic mice. A subset of plasmids containing c-DNA sequences of the indicated genes were denatured and immobilized on a nylon membrane. The filters were hybridized with a [$^{32}$P]-labeled cDNA, which was generated by reverse transcription of poly A+ mRNA from lung tissue of a non transgenic littermate, from a tumor nodule of the lung of a SP-C/*myc* transgenic and from a tumor nodule of the lung of a SP-C/*myc*/IgEGF double transgenic mouse. cycDI, cyclin DI

expression of these genes was distinctly increased in tumour nodules of SP-C/*myc*/IgEGF double transgenics, but not in tumours of SP-C/*myc* transgenics or in normal lung tissue (Figure 4). These preliminary results indicated increasing deregulation of the cell cycle at various stages of lung tumour development in the SP-C/*myc*/IgEGF double transgenics. It can be speculated that the deregulation of cell cycle regulating genes might be the reason for the decreased medial survival times in these mice (Figure 4).

### DISCUSSION

Constitutive overexpression of c-*myc* under the transcriptional control of the SP-C promoter is frequently associated with the development of bronchiolo-alveolar adenocarcinomas, adenomas or hyperplasias in transgenic mice. Hemizygous SPC/*myc* and SPC/IgEGF and homozygous SPC/*myc* transgenic mice examined in this study had a life span of between 9 and 14.25 months. Not all analysed transgenic mice developed bronchiolo-alveolar adenocarcinomas. We speculate that additional genetic changes have to occur for tumour induction, e.g. knock out of tumour suppressor and/or activation of proto-oncogenes. These events occur randomly and may explain that not all offspring develop tumours. However, death inducing spontaneous bronchiolo-alveolar adenocarcinomas are uncommon at this age, but it should considered, that spontaneous lung tumours are not a rare event in mice. Reported data are related to the age 24 month, are not specified for bronchiolo-alveolar entities and are not available for the hybrid strain CD2F1 (compare overview in Rittinghausen et al, 1997). It should be emphasized, that non-transgenic control mice of the breed and age used for transgenic studies, did not display any lung tumours. Therefore, it is evident, that the bronchiolo-alveolar neoplasias or hyperplasias were indeed caused by the overexpression of the c-*myc* transgene. The role of c-*myc* overexpression as a first step in the process of tumour formation was further confirmed by the gene dosage effect observed in homozygous transgenics, which showed accelerated tumour development in the lung. These findings support the hypothesis that this gene is causally involved in the development of human alveolar lung bronchiolo-alveolar adenocarcinomas, where overexpression of c-*myc* is frequently observed (Broers et al, 1993; Lorenz et al, 1994).

Overexpression of IgEGF under the control of the SP-C promoter led to the formation of hyperplasias of the alveolar epithelium in the lung of transgenic mice, whereas overexpression of TGFα in the lung of transgenic mice has been shown to induce enlarged parenchymal airspace and pulmonary fibrosis (Hardie et al, 1997). The induction of different phenotypes by IgEGF and TGFα might be due to the fact that EGF – but not TGFα – binds to other receptor subunits of the EGF receptor family; e.g. erbB2, 3 and/or 4 (Alimandi et al, 1997; Wang et al, 1998). A similar cooperation of c-*myc* and IgEGF, which led to accelerated bronchiolo- alveolar adenocarcinomas formation in SP-C/*myc*/IgEGF double transgenics was also demonstrated for hepatocarcinogenesis in transgenic mouse lines, which overexpress these oncogenes in hepatocytes (Tönjes et al, 1995).

First results indicate that other genes may be involved in the accelerated growth of tumors in SP-C/*myc*/IgEGF double transgenics (Figure 4). The expression level of cyclin D1 was shown to be strongly increased in tumour nodules of SP-C/*myc*/IgEGF double transgenic mice but not in lungs of non-transgenics or SP-C/*myc* transgenics. It is known that EGF induces cyclin D1

© 2001 Cancer Research Campaign expression (Ravitz et al, 1996; Ramljak et al, 1998), which is one of the most frequently overexpressed oncogenes in human bronchiolo-alveolar adenocarcinomas (Marchetti et al, 1998). Also, cdc2 and *c-jun* were overexpressed in lung tumours of SP-C/*myc*/IgEGF double transgenics. cdc2 is an important cell cycle controlling gene, which binds to and activates cyclin B1. Upregulation of *c-jun* was also observed in human cell lines established from NSCLC when stimulated by growth factors (Szabo et al, 1996). These observations indicate that the tumours in the transgenic mice are excellent models for human lung adenocarcinomas, which will be useful for understanding the molecular basis for the development of human lung cancer. Future experiments, involving gene expression profiles of developing tumours, that include a broader spectrum of tumour suppressor and oncogenes, will provide a more detailed view, which genes become involved during tumour progression in developing lung carcinomas in SP-C/*myc* as well as in SP-C/*myc*/IgEGF double transgenic mice.

The surfactant proteins SP-A, SP-B and SP-C were expressed moderately reduced or at similar levels in tumours of SP-C/*myc* and SP-C/*myc*/IgEGF transgenics as compared to lungs of non-transgenics indicating that bronchiolo-alveolar adenocarcinomas were derived from AT-II cells. Expression of SP-C was also shown to occur in human bronchiolo-alveolar adenocarcinomas (Linnoila et al, 1992) suggesting similarities between human bronchiolo-alveolar adenocarcinomas and the homologous tumour type in SP-C/*myc* transgenics. In summary we present a new model for bronchiolo-alveolar adenocarcinomas, which will be useful to address several questions about lung tumour formation.

### ACKNOWLEDGEMENTS

We thank Claudia Beyer for technical assistance. The authors are grateful to JA Whitsett for providing the SP-C-promoter DNA and c-DNA clones from SP-A, SP-B and SP-C. We gratefully acknowledge the support of this work by grants from the Deutsche Forschungsgemeinschaft (DFG) (III GK-GRK 139/3–99) and from the Deutsche Krebshilfe (10-0936-Ha I).

### REFERENCES

Alimandi M, Wang LM, Bottaro D, Lee CC, Kuo A, Frankel M, Fedi P, Tang C, Lippman M and Pierce JH (1997) Epidermal growth factor and betacellulin mediate signal transduction through co-expressed ErbB2 and ErbB3 receptors. *EMBO J* **15**: 5608–5617

Battista P, Pizzicannella G, Vitullo P, Palmirotta R and Mariani-Costantini R (1993) The epidermal growth factor family in pulmonary carcinoids: immunohistochemical evidence of growth-promoting circuits. *Mod Pathol* **6**: 162–166

Broers JL, Viallet J, Jensen SM, Pas H, Travis WD, Minna JD and Linnoila RI (1993) Expression of c-myc in progenitor cells of the bronchopulmonary epithelium and in a large number of non-small cell lung cancers. *Am J Respir Cell Mol Biol* **9**: 33–43

Dalemans W, Perraud F, Le-Meur M, Gerlinger P, Courtney M and Pavirani A (1990) Heterologous protein expression by transimmortalized differentiated liver cell lines derived from transgenic mice. *Biologicals* **18**: 191–198

Donaldson JC, Kaminsky DB and Elliott RC (1978) Bronchiolar carcinoma. Report of 11 cases and review of the literatur. *Cancer* **41**: 250–258

Facchini LM and Penn LZ (1998) The molecular role of Myc in growth and transformation: recent discoveries lead to new insights. *FASEB J* **12**: 633–651

Hardie WD, Bruno MD, Huelsman KM, Iwamoto HS, Carrigan PE, Leikauf GD, Whitsett JA and Korfhagen TR (1997) Postnatal lung function and morphology in transgenic mice expressing transforming growth factor-alpha. *Am J Pathol* **151**: 1075–1083

Hogan B, Beddingtin R, Costantini F and Lacy E (1994) *Manipulating the Mouse Embryo: A Laboratory Manual*. Cold Spring Harbor, NY, Cold Spring Harbor Laboratory

Korfhagen TR, Bruno MD, Glasser SW, Ciraolo PJ, Whitsett JA, Lattier DL, Wikenheiser KA and Clark JC (1992) Murine pulmonary surfactant SP-A gene: cloning, sequence, and transcriptional activity. *Am J Physiol* **263**: 546–554

Linnoila RI, Mulshine JL, Steinberg SM and Gazdar AF (1992) Expression of surfactant-associated protein in non-small-cell lung cancer: a discriminant between biologic subsets. *J Natl Cancer Inst Monogr* **13**: 61–66

Lorenz J, Friedberg T, Paulus R, Oesch F and Ferlinz R (1994) Oncogene overexpression in non-small-cell lung cancer tissue: prevalence and clinicopathological significance. *Clin Investig* **72**: 156–163

Marchetti A, Doglioni C, Barbareschi M, Buttitta F, Pellegrini S, Gaeta P, La Rocca R, Merlo G, Chella A, Angeletti CA, Dalla Palma P and Bevilacqua G (1998) Cyclin D1 and retinoblastoma susceptibility gene alterations in non-small cell lung cancer. *Int J Cancer* **75**: 187–192

Minna JD, Hippins GA and Glatstein EJ (1989) In: De Vita VT Jr., Hellman S and Rosenburg SA (eds) *Cancer: Principles and Practice of oncology*, Lippincott: Philadelphia, pp. 507–599

Moody TW (1996) Peptides and growth factors in non-small cell lung cancers. *Peptides* **17**: 545–555

Perez-Stable C, Altmann NH, Mehta PP, Deftos LJ and Roos BA (1997) Prostate cancer progression, metstasis, and gene expression in transgenic mice. *Cancer Res* **57**: 900–906

Ramljak D, Jones AB, Diwan BA, Perantoni AO, Hochadel JF and Anderson LM (1998) Epidermal growth factor and transforming growth factor-alpha-associated overexpression of cyclin D1, Cdk4, and c-Myc during hepatocarcinogenesis in *Helicobacter hepaticus*-infected A/JCr mice. *Cancer Res* **116**: 276–280

Ravitz MJ, Yan S, Dolce C, Kinniburgh AJ and Wenner CE (1996) Differential regulation of p27 and cyclin D1 by TGF-beta and EGF in C3H 10T1/2 mouse fibroblasts. *J Cell Physiol* **168**: 510–520

Rittinghausen S, Kaspareit J and Mohr U (1997) Incidence and spectrum of spontaneous neoplasms in Han: NMRI mice of both sexes. *Exp Toxicol Pathol* **49**: 347

Sandgren EP, Luetteke NC, Qiu TH, Palmiter RD and Brinster RL (1993) Transforming growth factor alpha dramatically enhances oncogene induced carcinogenesis in transgenic mouse pancreas and liver. *Mol Cell Biol* **13**: 320–330

Strayer MS, Guttantag SH and Ballard PL (1998) Targeting type II and Clara cells for adenovirus-mediated gene transfer using the surfactant protein B promoter. *Am J Respir Cell Mol Biol* **18**: 1–11

Szabo E, Riffe ME, Steinberg SM, Birrer MJ and Linnoila RI (1996) Altered cJUN expression: An early event in human lung carcinogenesis. *Cancer Res* **52**: 305–315

Tateishi M, Ishida T, Mitsudomi T, Kaneko S and Sugimachi K (1990) Immunohistochemical evidence of autocrine growth factors in adenocarcinoma of the human lung. *Cancer Res* **50**: 7077–7080

Tönjes RR, Lohler J, O'Sullivan JF, Kay GF, Schmidt GH, Dalemans W, Pavirani A and Paul D (1995) Autocrine mitogen IgEGF cooperates with c-myc or with the Hcs locus during hepatocarcinogenesis in transgenic mice. *Oncogene* **10**: 765–768

Tuveson DA and Jacks T (1999) Modeling human lung cancer in mice: similarities and shortcomings. *Oncogene* **18**: 5318–5324

Wang LM, Kuo A, Alimandi M, Veri MC, Lee CC, Kapoor V, Ellmore N, Chen XH, Pierce JH (1998) ErbB2 expression increases the spectrum and potency of ligand-mediated signal transduction through ErbB4. *Proc Natl Acad Sci* **95**: 6809–6814

Weaver TE and Whitsett JA (1991) Function and regulation of expression of pulmonary surfactant-associated proteins. *Biochem J* **273**: 249–264

Wikenheiser KA, Clark JC, Linnoila RI, Stahlmann MT and Whitsett JA (1992) Simian virus 40 large T antigen directed by transcriptional elements of the human surfactant protein C gene produces pulmonary adenocarcinomas in transgenic mice. *Cancer Res* **52**: 5342–5352

Yeh J and Yeh YC (1989) Transforming growth factor-alpha and human cancer. *Biomed Pharmacother* **43**: 651–659

*© 2001 Cancer Research Campaign*

## References

[PDF_00549] Alimandi M., Wang L. M., Bottaro D., Lee C. C., Kuo A., Frankel M., Fedi P., Tang C., Lippman M., Pierce J. H. (1997). Epidermal growth factor and betacellulin mediate signal transduction through co-expressed ErbB2 and ErbB3 receptors.. EMBO J.

[PDF_00553] Battista P., Pizzicannella G., Vitullo P., Palmirotta R., Mariani-Costantini R. (1993). The epidermal growth factor family in pulmonary carcinoids: immunohistochemical evidence of growth-promoting circuits.. Mod Pathol.

[PDF_00557] Broers J. L., Viallet J., Jensen S. M., Pass H., Travis W. D., Minna J. D., Linnoila R. I. (1993). Expression of c-myc in progenitor cells of the bronchopulmonary epithelium and in a large number of non-small cell lung cancers.. Am J Respir Cell Mol Biol.

[PDF_00561] Dalemans W., Perraud F., Le Meur M., Gerlinger P., Courtney M., Pavirani A. (1990). Heterologous protein expression by transimmortalized differentiated liver cell lines derived from transgenic mice (hepatomas/alpha 1 antitrypsin/ONC mouse).. Biologicals.

[PDF_00564] Donaldson J. C., Kaminsky D. B., Elliott R. C. (1978). Bronchiolar carcinoma: report of 11 cases and review of the literature.. Cancer.

[PDF_00566] Facchini L. M., Penn L. Z. (1998). The molecular role of Myc in growth and transformation: recent discoveries lead to new insights.. FASEB J.

[PDF_00568] Hardie W. D., Bruno M. D., Huelsman K. M., Iwamoto H. S., Carrigan P. E., Leikauf G. D., Whitsett J. A., Korfhagen T. R. (1997). Postnatal lung function and morphology in transgenic mice expressing transforming growth factor-alpha.. Am J Pathol.

[PDF_00579] Linnoila R. I., Mulshine J. L., Steinberg S. M., Gazdar A. F. (1992). Expression of surfactant-associated protein in non-small-cell lung cancer: a discriminant between biologic subsets.. J Natl Cancer Inst Monogr.

[PDF_00582] Lorenz J., Friedberg T., Paulus R., Oesch F., Ferlinz R. (1994). Oncogene overexpression in non-small-cell lung cancer tissue: prevalence and clinicopathological significance.. Clin Investig.

[PDF_00585] Marchetti A., Doglioni C., Barbareschi M., Buttitta F., Pellegrini S., Gaeta P., La Rocca R., Merlo G., Chella A., Angeletti C. A. (1998). Cyclin D1 and retinoblastoma susceptibility gene alterations in non-small cell lung cancer.. Int J Cancer.

[PDF_00593] Moody T. W. (1996). Peptides and growth factors in non-small cell lung cancer.. Peptides.

[PDF_00595] Perez-Stable C., Altman N. H., Mehta P. P., Deftos L. J., Roos B. A. (1997). Prostate cancer progression, metastasis, and gene expression in transgenic mice.. Cancer Res.

[PDF_00603] Ravitz M. J., Yan S., Dolce C., Kinniburgh A. J., Wenner C. E. (1996). Differential regulation of p27 and cyclin D1 by TGF-beta and EGF in C3H 10T1/2 mouse fibroblasts.. J Cell Physiol.

[PDF_00606] Rittinghausen S., Kaspareit J., Mohr U. (1997). Incidence and spectrum of spontaneous neoplasms in Han:NMRI mice of both sexes.. Exp Toxicol Pathol.

[PDF_00609] Sandgren E. P., Luetteke N. C., Qiu T. H., Palmiter R. D., Brinster R. L., Lee D. C. (1993). Transforming growth factor alpha dramatically enhances oncogene-induced carcinogenesis in transgenic mouse pancreas and liver.. Mol Cell Biol.

[PDF_00613] Strayer M. S., Guttentag S. H., Ballard P. L. (1998). Targeting type II and Clara cells for adenovirus-mediated gene transfer using the surfactant protein B promoter.. Am J Respir Cell Mol Biol.

[PDF_00616] Szabo E., Riffe M. E., Steinberg S. M., Birrer M. J., Linnoila R. I. (1996). Altered cJUN expression: an early event in human lung carcinogenesis.. Cancer Res.

[PDF_00619] Tateishi M., Ishida T., Mitsudomi T., Kaneko S., Sugimachi K. (1990). Immunohistochemical evidence of autocrine growth factors in adenocarcinoma of the human lung.. Cancer Res.

[PDF_00625] Tuveson D. A., Jacks T. (1999). Modeling human lung cancer in mice: similarities and shortcomings.. Oncogene.

[PDF_00622] Tönjes R. R., Löhler J., O'Sullivan J. F., Kay G. F., Schmidt G. H., Dalemans W., Pavirani A., Paul D. (1995). Autocrine mitogen IgEGF cooperates with c-myc or with the Hcs locus during hepatocarcinogenesis in transgenic mice.. Oncogene.

[PDF_00627] Wang L. M., Kuo A., Alimandi M., Veri M. C., Lee C. C., Kapoor V., Ellmore N., Chen X. H., Pierce J. H. (1998). ErbB2 expression increases the spectrum and potency of ligand-mediated signal transduction through ErbB4.. Proc Natl Acad Sci U S A.

[PDF_00631] Weaver T. E., Whitsett J. A. (1991). Function and regulation of expression of pulmonary surfactant-associated proteins.. Biochem J.

[PDF_00633] Wikenheiser K. A., Clark J. C., Linnoila R. I., Stahlman M. T., Whitsett J. A. (1992). Simian virus 40 large T antigen directed by transcriptional elements of the human surfactant protein C gene produces pulmonary adenocarcinomas in transgenic mice.. Cancer Res.

[PDF_00637] Yeh J., Yeh Y. C. (1989). Transforming growth factor-alpha and human cancer.. Biomed Pharmacother.

